# Suicidal wrist bites

**DOI:** 10.1007/s12024-023-00769-1

**Published:** 2024-02-28

**Authors:** Claas Buschmann

**Affiliations:** Institute of Legal Medicine, University Hospital Schleswig-Holstein, Arnold-Heller-Str. 3, Building 28, 24105 Kiel, Germany

**Keywords:** Suicide, Sharp force, Bite marks, Autopsy

## Abstract

Wrist injuries are not uncommon in forensic routine and are usually found in the context of suicides or as a result of psychiatric illnesses, e.g., borderline disorders. Sharp objects (knives, broken glass, etc.) are usually used. In the case reported here, a paranoid-schizophrenic man not only injured himself with razor blades on both wrists, but he also inflicted extensive wrist bite injuries using his dental prosthesis. In addition to the severance of flexor tendons, venous vessels and the left radial artery were torn with subsequent blood loss. At the time of death, there was also acute exposure to methadone and opiates. Patients suffering from psychotic illnesses have an increased risk of committing spectacular or bizarre suicides.

## Case report

A 61-year-old man was found dead at night in the stairwell of the apartment building he lived in by a neighbor after the neighbor heard a “wolf-like growl” and went to investigate. The body showed extensive injuries on both wrists. From where the body was found in the stairwell, traces of blood ran through the open apartment door into the apartment’s bathroom. Large areas of blood, two bloody razor blades, and a denture with chunks of flesh were found there. A pack of “ibuprofen” (NSAID, 400 mg) was also detected in the apartment. Five tablets were missing. A suicide note was not found.

The man was an “artist,” smoked heavily, and abused illicit drugs for decades. According to the neighbors, he had recently exhibited schizophrenic traits and was apparently suffering from paranoia. Yet there were no hints for medical treatment.

### Post-mortem examination

**Fig. 1:**
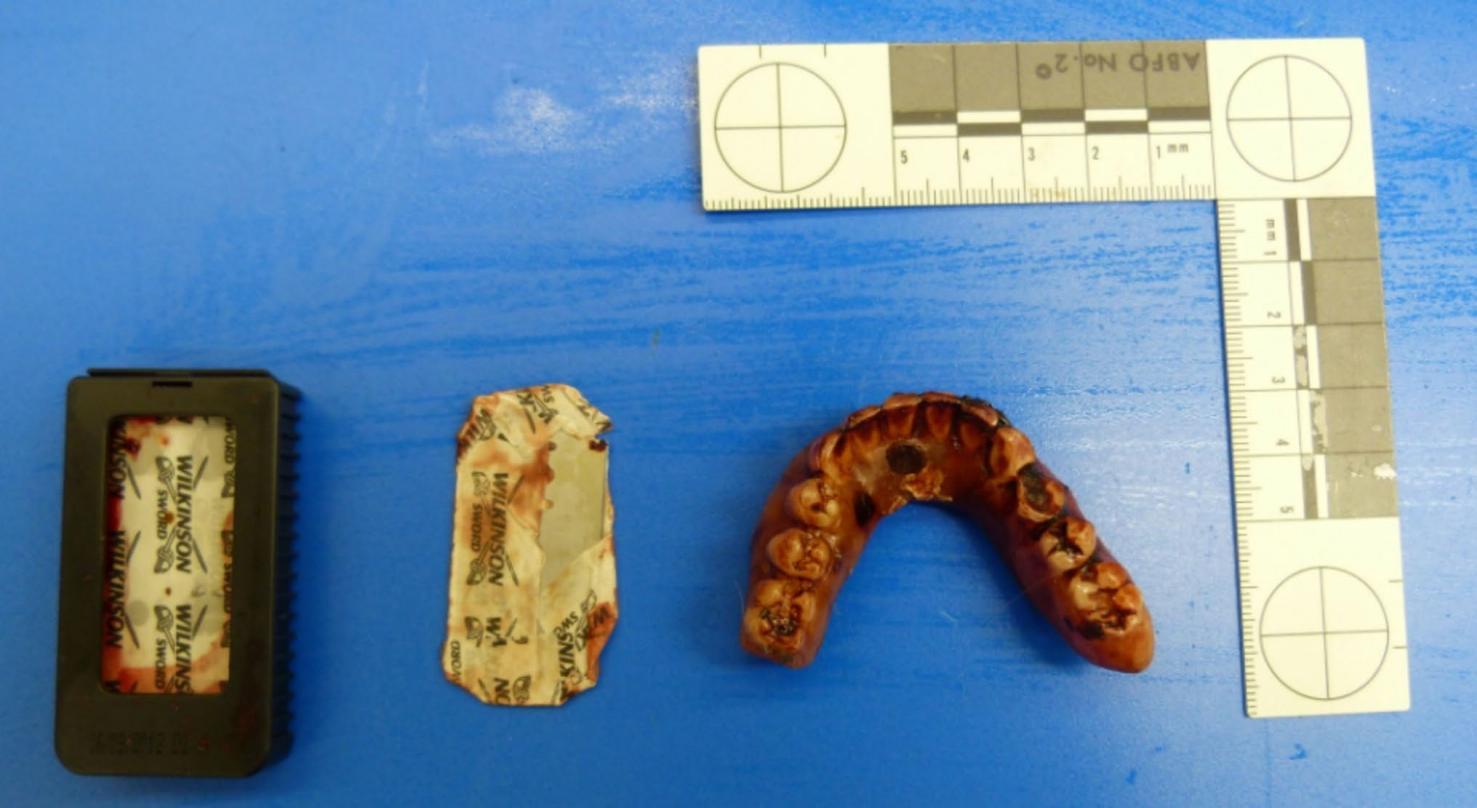
Bloodied sharp razor blades and bloodied lower jaw prosthesis

**Fig. 2:**
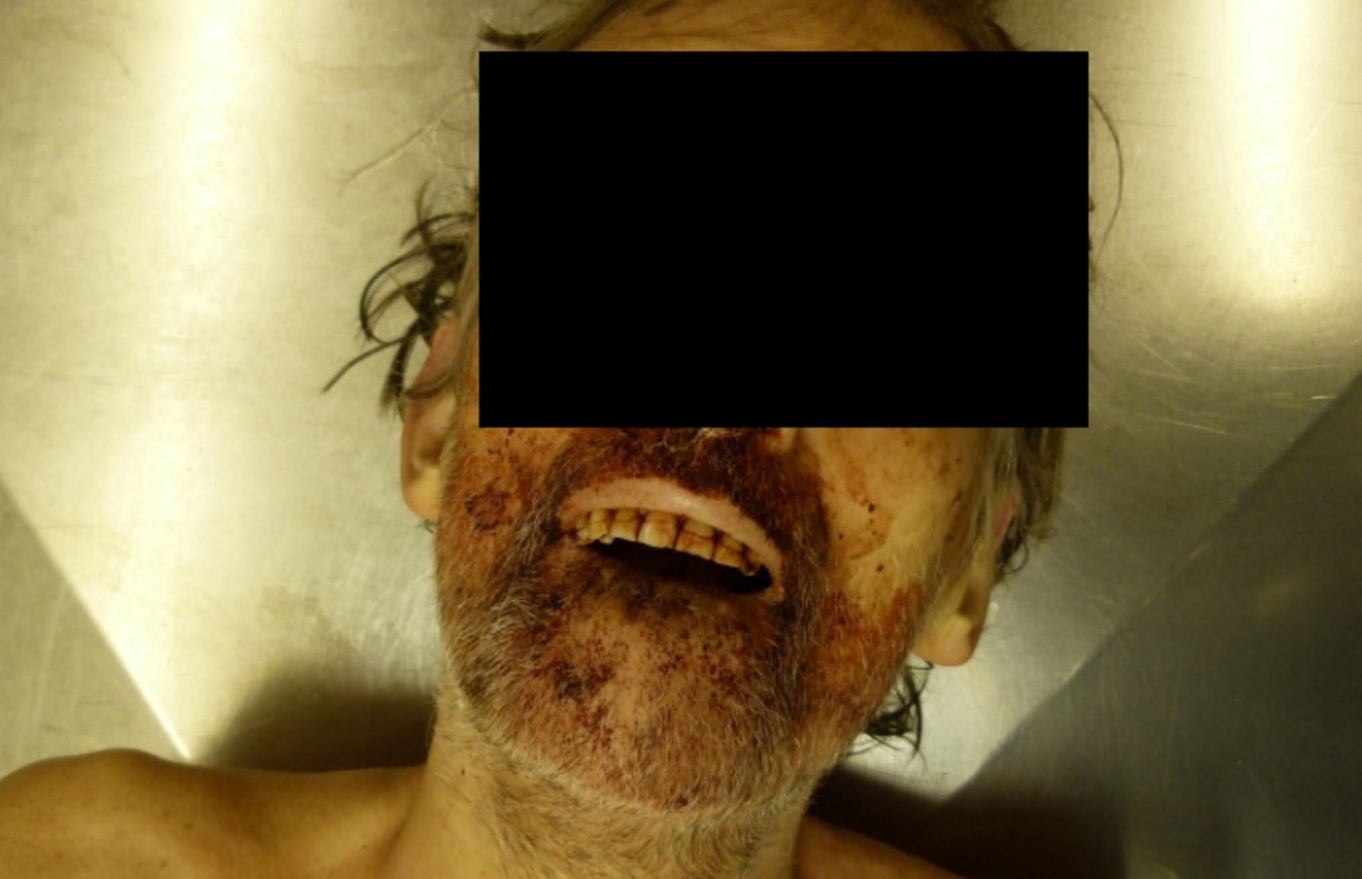
Face and front teeth smeared with blood

Together with the pale body, two bloodied sharp razor blades and a bloodied lower jaw prosthesis with dried soft tissue–like adhesions were presented at post-mortem examination. Modifications on the prosthesis such as sharpening or filing were not noticeable (Fig. 1). The facial skin in the nose and mouth area was smeared with blood, as were the (own) front teeth (Fig. 2). Some metal pin teeth were implanted in the upper jaw.

**Fig. 3:**
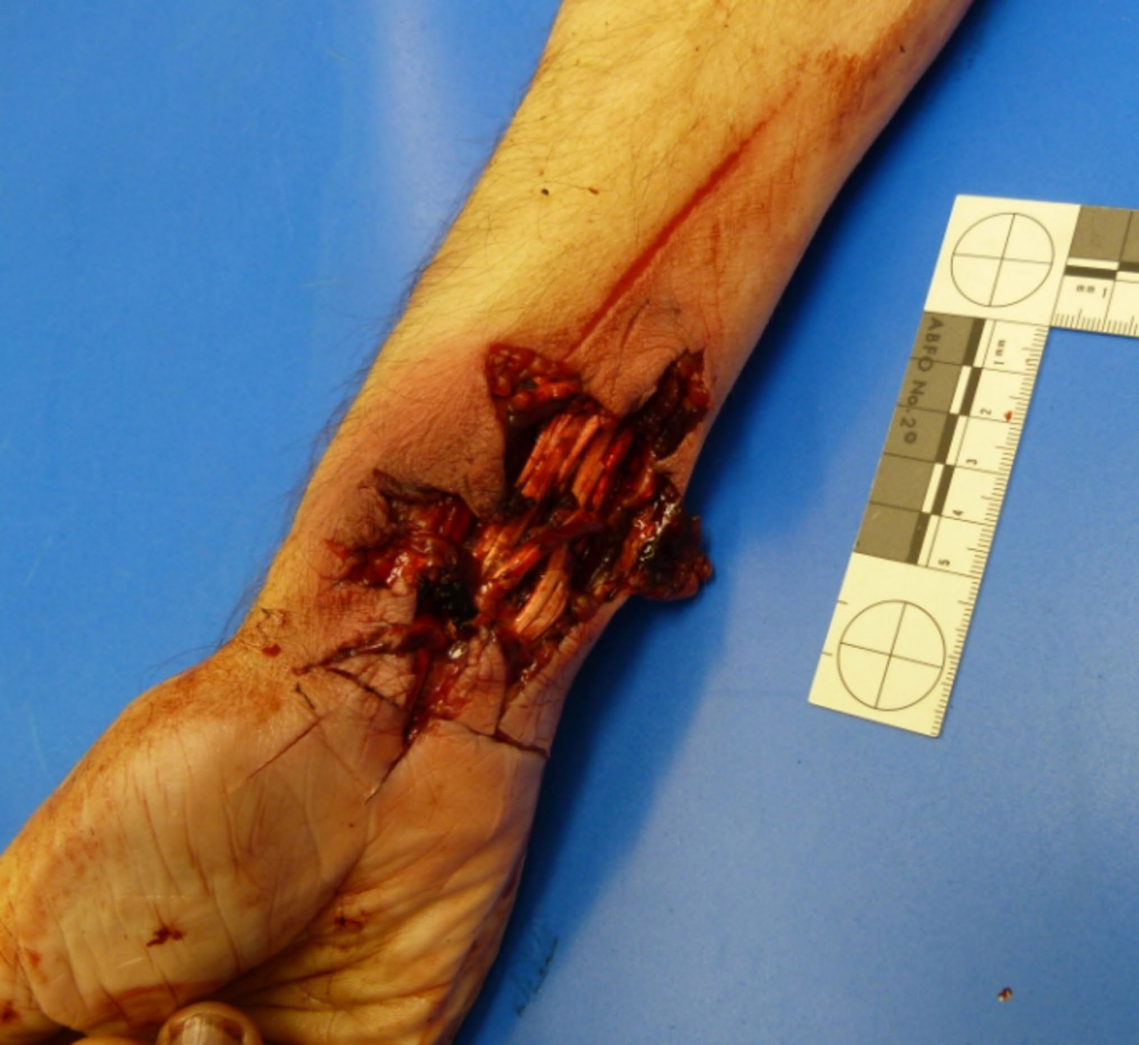
Right wrist

**Fig. 4:**
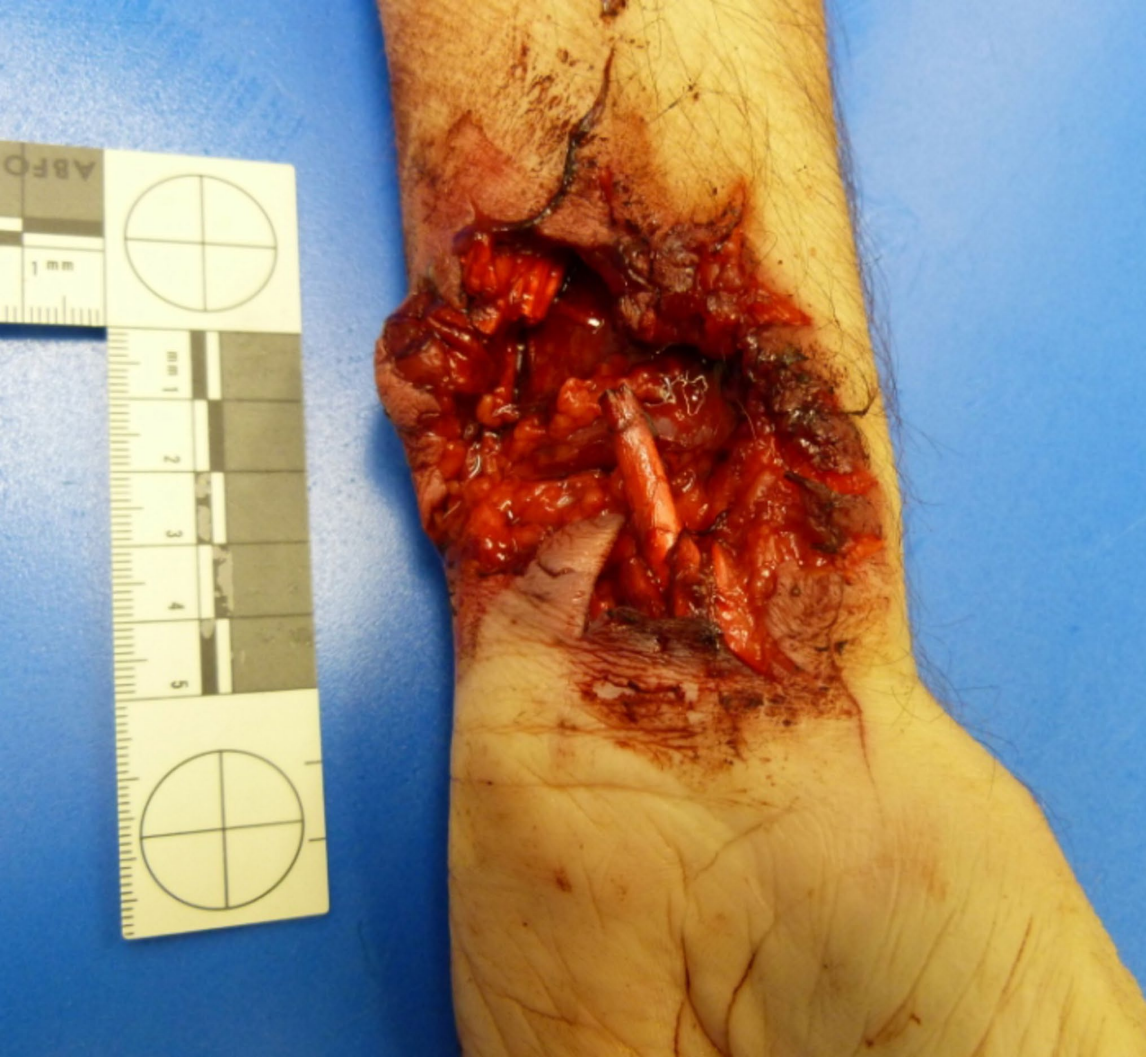
Left wrist

On the inside of the right wrist, there was a deep injury that appeared to be mangled measuring approximately 5 × 4 cm, as well as a superficial, longitudinal skin incision approximately 15 cm long and further transverse, deeper skin incisions approximately 4 cm long with smooth edges of the wound with surrounding hesitation marks (Fig. 3). On the left wrist, there was another deep flesh wound, approximately 5 × 5 cm in size, which appeared to have been mangled, with hesitation marks surrounding it (Fig. 4).

### Autopsy

On the left wrist, there was tearing of all flexor tendons and gross tears of the superficial skin veins. The left radial artery was roughly severed and torn. On the right wrist, the superficial flexor tendons were severed, and the large skin veins were extensively torn. In addition to the signs of severe blood loss (sparse livor mortis, pale internal organs), there was evidence of poisoning at the time of death with an enlarged urinary bladder (500 ml urine) as well as edema of the lungs and brain. The remaining organ findings were age-appropriate. There were no soft tissue particles in the stomach. Cause of death was hemorrhagic shock.

Toxicological analysis revealed that opiates and methadone had been ingested close before death, but not in toxic-lethal levels. NSAID was not detected, and there was no alcoholic influence at the time of death. Hair analysis showed that the man had frequently consumed methadone and heroin as well as occasional cocaine, amphetamine, buprenorphine, and benzodiazepines in the last few months prior to death.

## Discussion

Human bite injuries inflicted by others are occasionally examined in clinical forensic medicine, particularly in the context of sexual offenses or in children [1–4]. Self-inflicted bite injuries are rare [5]. They can occur not only as self-harm but also as an emotional reaction to pain or as a counterstimulus to relieve pain [6]. However, suicidal bites are an absolute rarity [7, 8].

The reported case supports the well-known finding that patients suffering from psychotic illnesses have an increased risk of committing spectacular or bizarre suicides, which in this case is also a particularly painful type of self-harm. It should be noted that analgetics were not detected in the toxicological analysis—but opiates and opioids have pain-killing potential. However, schizophrenic patients are reported to have a reduced sense of pain [9]. Whether the reported psychiatric symptoms were due to an endogenous psychosis, an exogenous psychosis (due to long-term drug abuse) or a mixed form could not be clarified retrospectively.

In the present case, there was a mixture of cuts and bites on both wrists. While razor blades were initially used (superficial hesitation cuts, sharp severing of tendons), the suicidal act apparently escalated quite quickly and resulted in the biting out of muscles and the severing of the left radial artery, which was clearly caused by a tear/bite, with corresponding blood loss. This is to be distinguished from so-called autocannibalism, as the pieces of tissue bitten out were not consumed [10]. It remains open whether the discovery of the body in the stairwell might be interpreted as a cry for help.

Once acute suicidality has been diagnosed, e.g., in prison inmates or patients in psychiatric wards, in addition to appropriate medical interventions, other extensive suicide prevention measures are regularly taken. Nevertheless, the reported case shows once again that not every suicide can be prevented. Suicides by self-inflicted bites are possible, especially in the context of psychotic illnesses.
